# Interleukin-10 Facilitates Glutamatergic Synaptic Transmission and Homeostatic Plasticity in Cultured Hippocampal Neurons

**DOI:** 10.3390/ijms20133375

**Published:** 2019-07-09

**Authors:** Miroslav N. Nenov, Maxim V. Konakov, Ilia Y. Teplov, Sergey G. Levin

**Affiliations:** 1 Institute of Theoretical and Experimental Biophysics of the Russian Academy of Sciences, Pushchino, Moscow 142290, Russia; 2 Alzheimer’s Center at Temple, Lewis Katz School of Medicine, Temple University, Philadelphia, PA 19140, USA

**Keywords:** Interleukin-10, mEPSC, excitatory glutamatergic synaptic transmission, homeostatic synaptic plasticity, primary hippocampal culture

## Abstract

Anti-inflammatory cytokines are known to exert neuroprotective action ameliorating aberrant neuronal network activity associated with inflammatory responses. Yet, it is still not fully understood if anti-inflammatory cytokines play a significant role in the regulation of synaptic activity under normal conditions. Thus, the aim of our study was to investigate the effect of Interleukin-10 (IL-10) on neuronal synaptic transmission and plasticity. For this we tested the effect of IL-10 on miniature excitatory postsynaptic currents (mEPSC) and intracellular Ca^2+^ responses using whole-cell patch clamp and fluorescence microscopy in 13–15 DIV primary hippocampal neuroglial culture. We found that IL-10 significantly potentiated basal glutamatergic excitatory synaptic transmission within 15 min after application. Obtained results revealed a presynaptic nature of the effect, as IL-10 in a dose-dependent manner significantly increased the frequency but not the amplitude of mEPSC. Further, we tested the effect of IL-10 on mEPSC in a model of homeostatic synaptic plasticity (HSP) induced by treatment of primary hippocampal culture with 1 µM of tetrodotoxin (TTX) for a 24 h. It was found that 15 min application of IL-10 at established HSP resulted in enhanced mEPSC frequency, thus partially compensating for a decrease in the mEPSC frequency associated with TTX-induced HSP. Next, we studied if IL-10 can influence induction of HSP. We found that co-incubation of IL-10 with 1 µM of TTX for 24 h induced synaptic scaling, significantly increasing the amplitude of mEPSC and Ca^2+^ responses to application of the AMPA agonist, 5-Fluorowillardiine, thus facilitating a compensatory postsynaptic mechanism at HSP condition. Our results indicate that IL-10 potentiates synaptic activity in a dose- and time-dependent manner exerting both presynaptic (short-term exposure) and postsynaptic (long-term exposure) action. Obtained results demonstrate involvement of IL-10 in the regulation of basal glutamatergic synaptic transmission and plasticity at normal conditions.

## 1. Introduction

Interleukin-10 (IL-10) is an anti-inflammatory cytokine which plays an important role in the regulation of neuronal function under pathophysiological conditions associated with elevated neuro-inflammatory response for different neurodegenerative and psychiatric disorders [1,2]. It has been shown that IL-10 can exert neuroprotective action at hypoxic and ischemic conditions as well as lipopolysaccharide induced neuro-inflammation [3,4,5]. A beneficial effect of IL-10 gene therapy on neuronal function was found in neuropathic pain models and animal models for neurodegenerative disease [1,6,7,8]. Neuroprotective effects of IL-10 are associated, in particular, with the modulation of voltage-gated ion channels and ionotropic receptors such as AMPA and NMDA receptors via posttranslational modifications and the regulation of their expression levels [9,10,11,12,13,14,15]. Although, there are several studies providing thorough information on the pharmacology and function of IL-10 in the regulation of neuronal excitability and synaptic activity under pathophysiological conditions, little is known about the role of this anti-inflammatory cytokine in the regulation of neuronal network activity under physiological conditions. Recently, it has been shown that exogenous IL-10 can regulate GABAergic inhibitory synaptic transmission in a dose-dependent manner [16], however, IL-10 action on glutamatergic excitatory synaptic transmission and neuronal network plasticity remains elusive. In the present study, using whole-cell patch clamp electrophysiology and [Ca^2+^]_i_ imaging fluorescence microscopy we aimed to study the effect of exogenously added IL-10 on basal glutamatergic excitatory synaptic transmission and homeostatic synaptic plasticity in cultured hippocampal neurons.

## 2. Results

### 2.1. Effect of Interleukin-10 on Basal Glutamatergic Synaptic Transmission

**Figure 1:**
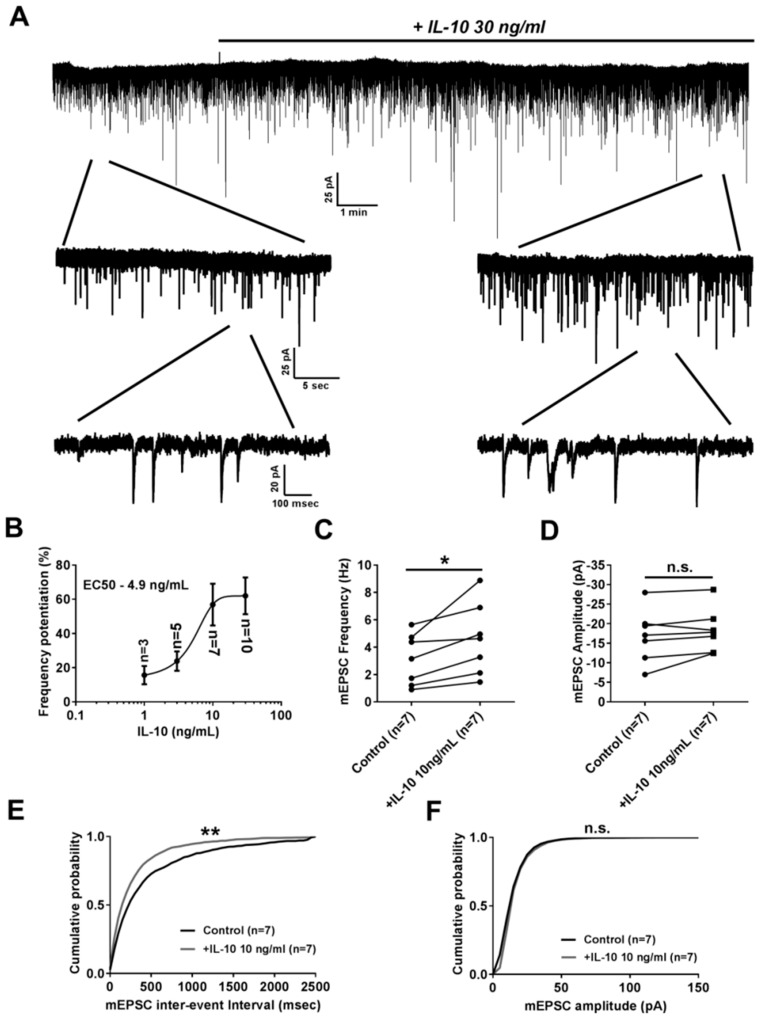
IL-10 presynaptically facilitates basal glutamatergic synaptic transmissions. (**A**) Representative traces of miniature excitatory postsynaptic currents (mEPSC) presented at different time scales. Exposure to IL-10 is indicated with a solid line. (**B**) Dose-dependent curve of IL-10 action on mEPSC frequency. (**C**) Graphical plot representing mEPSC frequency before and 15 min after IL-10 application. (**D**) Graphical plot representing mEPSC amplitude before and 15 min after IL-10 application. (**E**) and (**F**) Cumulative probability distribution plots representing mEPSC inter-event interval (**E**) and amplitude (**F**) distributions before and 15 min after IL-10 application. * *p* < 0.05 with paired *t*-test; ** *p* < 0.01 with Kolmogorov–Smirnov test.

To study the effect of IL-10 on glutamatergic excitatory synaptic transmission we tested how 15 min application of this anti-inflammatory cytokine affects frequency and amplitude of miniature excitatory postsynaptic currents (mEPSC) in neurons from primary hippocampal neuro-glial culture (Figure 1A). It was found that 15 min application of IL-10 at different concentrations resulted in a dose-dependent increase of mEPSC frequency with an EC50 value of 4.9 ng/mL (Figure 1B). For instance, 10 ng/mL of IL-10 significantly increased mEPSC frequency, as was found for averaged values (4.59 ± 0.99 Hz after IL-10 application, *n* = 7 vs. 3.11 ± 0.71 Hz before application in control, *n* = 7; *p* < 0.05 with paired *t*-test, Figure 1C) and for the analysis of mEPSC frequency probability distribution (*p* < 0.01 with Kolmogorov–Smirnov test, Figure 1E). At the same time there was no effect of IL-10 on the amplitude of mEPSC. For example, 10 ng/mL of IL-10 did not show any significant effect on mEPSC amplitude neither for averaged values (−18.27 ± 2.11 pA after IL-10 application, *n* = 7 vs. −16.92 ± 2.54 pA before application in control, *n* = 7; *p* > 0.05 with paired *t*-test, Figure 1D) nor for the mEPSC amplitude probability distribution (*p* > 0.05 with Kolmogorov–Smirnov test, Figure 1F).

### 2.2. Effects of Interleukin-10 on Homeostatic Synaptic Plasticity

**Figure 2:**
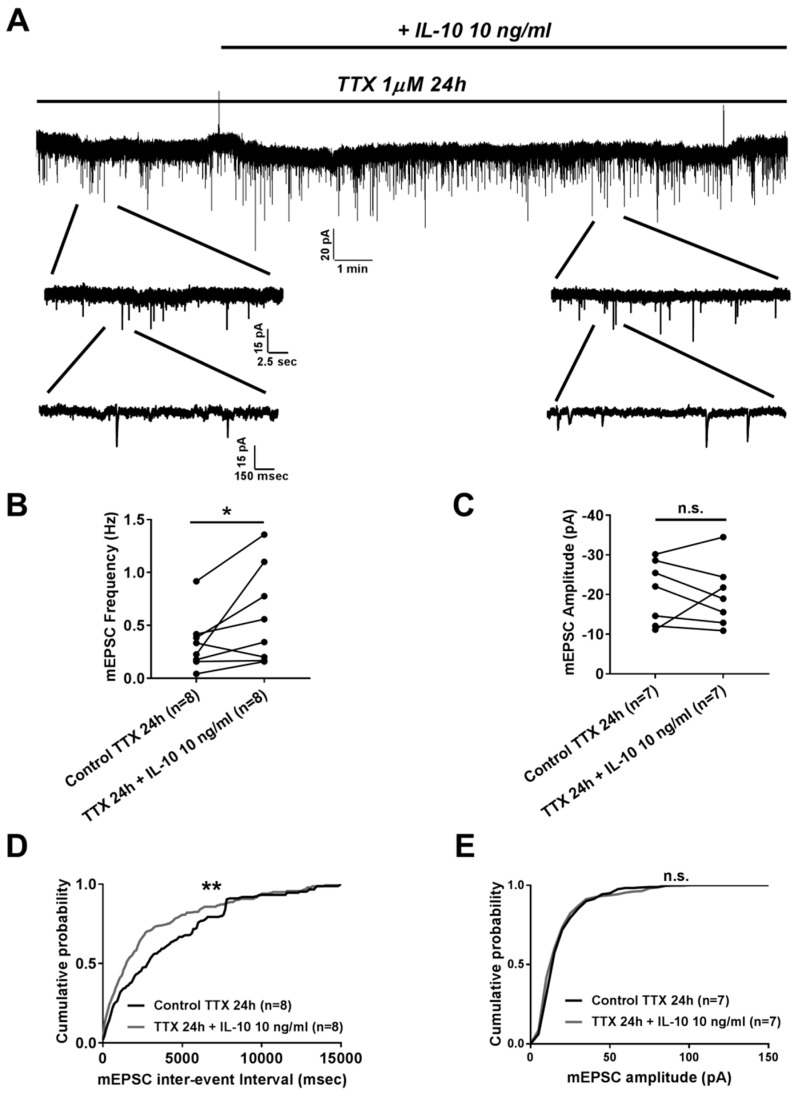
Application of IL-10 exerts presynaptic effect on homeostatic synaptic plasticity modeled with 1 µM TTX treatment for 24 h. (**A)** Representative traces of mEPSC presented at different time scales. Exposure to IL-10 is indicated by the solid line. (**B**) Graphical plot representing mEPSC frequency before and 15 min after Il-10 application. (**C**) Graphical plot representing mEPSC amplitude before and 15 min after IL-10 application. (**D**) and (**E**) Cumulative probability distribution plots representing mEPSC inter-event interval (D) and amplitude (E) distribution before and 15 min after IL-10 application. * *p* < 0.05 with paired *t*-test; ** *p* < 0.01 with Kolmogorov–Smirnov test.

Further we studied the effect of IL-10 on homeostatic plasticity by measuring frequency and amplitude of mEPSC from neurons that were incubated with 1 µM of tetrodotoxin (TTX) for 24 h prior to the experiment. It was found that, similarly to its effect on basal synaptic transmission, IL-10 added for 15 min was able to increase frequency of mEPSC with no effect on its amplitude (Figure 2A). Thus, 10 ng/mL of IL-10 significantly increased mEPSC frequency, as it was found for averaged values (0.58 ± 0.16 Hz after IL-10 application, *n* = 8 vs. 0.33 ± 0.09 Hz before application in 24 h TTX-treated group, *n* = 8; *p* < 0.05 with paired *t*-test, Figure 2B) and for the analysis of mEPSC frequency probability distribution (*p* < 0.01 with Kolmogorov–Smirnov test, Figure 2D). At the same time 10 ng/mL of IL-10 did not show any significant effect on mEPSC amplitude neither for averaged values (−19.85 ± 3.03 pA after IL-10 application, *n* = 7 vs. 20.59 ± 2.99 pA before application in 24 h TTX-treated group, *n* = 7; *p* > 0.05 with paired *t*-test, Figure 2C) nor for mEPSC amplitude probability distribution (*p* > 0.05 with Kolmogorov–Smirnov test, Figure 2E).

**Figure 3:**
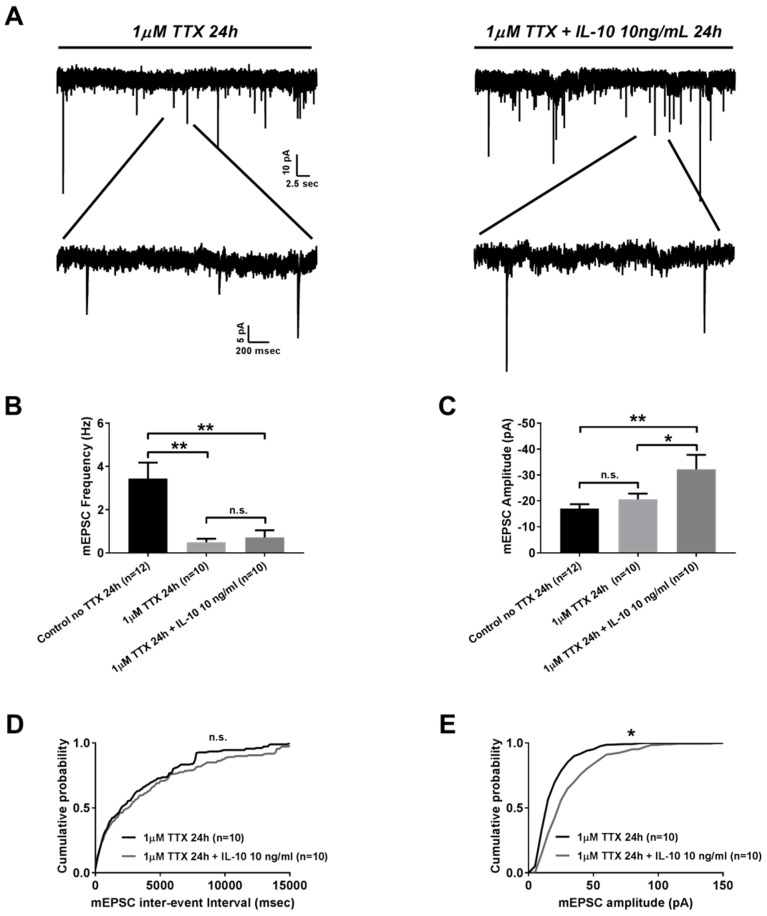
Co-treatment with IL-10 facilitates synaptic scaling in a model of homeostatic synaptic plasticity induced with 1 µM tetrodotoxin (TTX) treatment for 24 h. (**A**) Representative traces of mEPSC treated for 24 h ether with 1 µM TTX alone or 1 µM TTX co-incubated with 10 ng/mL IL-10. Traces are presented at different time scales. Each condition is highlighted with a solid line. (**B**) Graphical bars representing mEPSC frequency in control and at 24 h treatment with 1 µM TTX or 1 µM TTX co-incubated with 10 ng/mL of IL-10. (**C**) Graphical bars representing mEPSC amplitude in control and at 24 h treatments with 1 µM TTX or 1 µM TTX co-incubated with 10 ng/mL of IL-10. (**D**) and (**E**) Cumulative probability distribution plots representing mEPSC inter-event interval (**D**) and amplitude (**E**) distribution in neurons treated for 24 h with 1 µM TTX or 1 µM TTX co-incubated with 10 ng/mL of IL-10. * *p* < 0.05 or ** *p* < 0.01 with ANOVA followed by a Bonferroni posthoc test; * *p* < 0.05 with Kolmogorov–Smirnov test (for **E**).

Next we studied whether IL-10 is capable of exerting any effect on the formation of homeostatic plasticity. For this we compared frequency and amplitude of mEPSC from neurons incubated for 24 h with 1 µM of TTX alone to those of neurons that in addition to 1 µM of TTX, were co-incubated with 10 ng/mL of IL-10 for the same period of time (Figure 3A). A 24 h incubation period with TTX resulted in a significant reduction of mEPSC frequency compared to the values obtained from the control mEPSC recorded from neurons without 24 h incubation with TTX (3.44 ± 0.74 Hz in control, *n* = 12 vs. 0.49 ± 0.16 Hz in 24 h TTX-treated group, *n* = 10; *p* < 0.01 with one-way ANOVA followed by a Bonferroni posthoc test, Figure 3B). Unlike 15 min application, 24 h co-incubation with 10 ng/mL of IL-10 did not affect mEPSC frequency (3.44 ± 0.74 Hz in control, *n* = 12 vs. 0.71 ± 0.33 Hz in 24 h TTX + 10 ng/mL Il-10-treated group, *n* = 10; *p* < 0.01 with one-way ANOVA followed by a Bonferroni posthoc test, Figure 3B), and was not be able to significantly reverse the phenotype observed for 24 h incubation with TTX alone (0.49 ± 0.16 Hz in 24 h TTX-treated group, *n* = 10 vs. 0.71 ± 0.33 Hz in 24 h TTX + 10 ng/mL IL-10-treated group, *n* = 10; *p* > 0.05 with one-way ANOVA followed by a Bonferroni posthoc test, Figure 3B). The insignificance of mEPSC frequency between neurons treated with TTX and neurons treated with combination of TTX and IL-10 could also be seen from the analysis of mEPSC frequency cumulative distribution (Figure 3D). HSP induced by 24 h incubation with TTX is known to be associated not only with reduction in mEPSC frequency, but also with changes in mEPSC amplitude which reflects plastic adaptation to a drop in mEPSC frequency, known as the synaptic scaling of neuronal network. When we compared the mEPSC amplitude of 24 h TTX-treated neurons to the untreated control, we found a noticeable, yet, insignificant increase in the mEPSC amplitude (−17.07 ± 1.64 pA in control, *n* = 12 vs. −20.61 ± 2.19 pA in 24 h TTX-treated group, *n* = 10; *p* > 0.05 with one-way ANOVA followed by a Bonferroni posthoc test, Figure 3C). Co-incubation with IL-10 resulted in the further increase of mEPSC amplitude which, in this case, was significantly different from the non-TTX treated control (−17.07 ± 1.64 pA in control, *n* = 12 vs. -32.17 ± 5.6 pA in 24 h TTX + 10 ng/mL IL-10-treated group, *n* = 10; *p* < 0.01 with one-way ANOVA followed by a Bonferroni posthoc test, Figure 3C) as well as from neurons that were treated with TTX alone (-20.61 ± 2.19 pA in 24 h TTX-treated group, *n* = 10 vs. -32.17 ± 5.6 pA in 24 h TTX + 10 ng/mL IL-10-treated group, *n* = 10; *p* < 0.05 with one-way ANOVA followed by a Bonferroni posthoc test, Figure 3C). Significance in the increase of mEPSC amplitude between neurons treated with TTX alone and neurons treated with combination of TTX and IL-10 was also observed in the analysis of the mEPSC amplitude cumulative distributions (Figure 3E).

### 2.3. Effect of IL-10 on AMPA Receptor Agonist 5-Fluorowillardiine-Induced Neuronal Ca^2+^ Responses at Condition of Homeostatic Synaptic Plasticity

**Figure 4:**
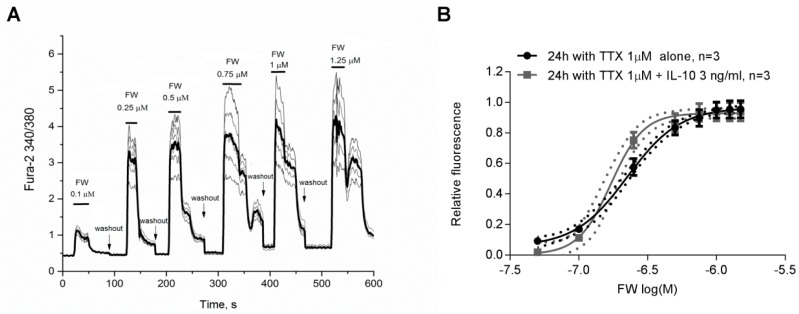
Co-treatment with IL-10 increases dose response action of 5-Fluorowillardiine (FW) on Ca^2+^ signals in cultured hippocampal neurons. (**A**) Representative trace showing [Ca^2+^]_i_ responses in Fura-2 loaded neurons induced with application of FW at gradually increasing concentrations. Solid black line represents averaged signal from a group of neurons. Ca^2+^ signals from individual neurons are represented by the gray thin lines. (**B**) Dose-dependent curves of FW action on Ca^2+^ signals in neurons treated with 1 µM of TTX for 24 h (black curve) or neurons treated with a combination of 1 µM TTX and 3 ng/mL of IL-10 for 2 h (gray curve).

Finally, we decided to test if the increase in mEPSC amplitude induced by treatment with TTX and IL-10 for a 24 h co-incubation period is associated with changes in AMPA receptor activity. For this we studied the dose-dependent effect of the AMPA receptor selective agonist 5-Fluorowillardiine on intracellular Ca^2+^ signals ([Ca^2+^]_i_) in Fura-2 loaded neurons (Figure 4A) that were incubated with either TTX alone or TTX and 3 ng/mL of IL-10 for 24 h. We found that co-incubation with IL-10 significantly increased [Ca^2+^]_i_ responses to application of 5-Fluorowillardiine at different concentrations resulting in a leftward shift of the dose-dependent curve which was accompanied by significant changes in EC50 (0.23 ± 0.01 µM in 24 h TTX-treated group, *n* = 3 vs. 0.17 ± 0.002 µM in in 24 h TTX+10 ng/mL IL-10-treated group, *n* = 3; *p* < 0.05 with student *t*-test, Figure 4B) and Hill coefficient (2.35 ± 0.2 in 24 h TTX-treated group, *n* = 3 vs. 3.65 ± 0.1 in 24 h TTX+10 ng/mL IL-10-treated group, *n* = 3; *p* < 0.05 with student *t*-test, Figure 4B).

## 3. Discussion

In the present study we found that exogenously added IL-10 effectively modulates mEPSC in pyramidal neurons from primary neuroglial hippocampal culture. IL-10 regulates excitatory glutamatergic synaptic transmission at basal level, and during plastic changes associated with a blockade of neuronal excitability, resulting in synaptic scaling.

Recently it has been found that IL-10 affects GABAergic synaptic transmission. Suryanarayanan and coauthors showed that the application of IL-10 exerted both presynaptic and postsynaptic effects decreasing frequency and amplitude of miniature inhibitory postsynaptic currents (mIPSC) in a dose-dependent manner. Reduction in both mIPSC frequency and amplitude was evident within several minutes after IL-10 administration [16]. In the present study, we found that opposite to its effect on inhibitory GABAergic synaptic transmission, IL-10 significantly potentiates excitatory glutamatergic synaptic transmission. In this case IL-10 acts specifically via presynaptic mechanisms. Thus, application of IL-10 within several minutes increased mEPSC frequency in a dose-dependent manner with no effect on mEPSC amplitude. Our results further emphasize on the importance of IL-10 in the regulation of basal synaptic activity and show that presynaptic effects of IL-10 might be a dominant mechanism for modulation of both excitatory and inhibitory basal synaptic transmission by this anti-inflammatory cytokine. Yet, the exact mechanism associated with a difference in IL-10 presynaptic action on GABAergic and glutamatergic transmission requires additional investigation.

Our results are in line with previously published observations showing the importance of cytokines in the regulation of basal excitatory glutamatergic synaptic transmission. It was found that pro-inflammatory cytokines such as TNF-α and IL-18 effectively enhance basal synaptic activity increasing both mEPSC frequency and amplitude [17,18]. On the other hand, pro-inflammatory cytokines such as IL-17 and IL-6 were found to act as limiting factors for basal excitatory synaptic transmission. IL-17 KO mice showed an increase in both mEPSC frequency and amplitude, and genetically modified mice with impaired IL-6 trans-signaling showed an increase in mEPSC frequency [19,20]. Another pro-inflammatory cytokine well known for its role in the regulation of synaptic plasticity, IL-1β, was found to show dose-dependent inhibitory presynaptic action on basal glutamatergic synaptic transmission by decreasing mEPSC frequency in hippocampal cultured neurons [21]. Previously it was found that IL-10 can occlude negative effects of IL-1β on long-term plasticity [22]. Our results indicate that IL-10 may exert its anti-inflammatory effects by antagonizing the negative presynaptic effect of IL-1β on glutamatergic synaptic transmission via potentiation of presynaptic glutamate release. Yet, the exact role of IL-10 in the regulation of basal synaptic transmission in context of its interplay with other cytokines is a subject of further investigation.

Finally, we found that IL-10 can play an important modulatory role in the synaptic scaling associated with the inhibition of neuronal firing in a model of homeostatic synaptic plasticity (HSP) induced with 24 h TTX treatment. In our experiments with DIV 13–15 neuroglial culture, treatment with TTX for 24 h resulted in a significant reduction in mEPSC frequency. At the same time we did not find any significant changes in the mEPSC amplitude of 24 h TTX treated neurons which can be also observed in other studies using neuronal cultures of similar age and/or similar duration for TTX treatment [23,24]. Next, we found that the short-term application of IL-10 to established HSP, similar to its effect on basal glutamatergic synaptic transmission, significantly increased mEPSC frequency partially compensating for the reduction of mEPSC frequency induced with 24 h TTX treatment. Further, we tested whether IL-10 can exert any effect on HSP induction. It was found that when co-incubated with TTX for 24 h, IL-10 favors synaptic scaling as manifested by increased mEPSC amplitude. Previously it has been found that the inflammatory cytokine, TNF-α, is critical for synaptic scaling. Secreted by glia in response to prolonged deprivation of neuronal activity with 48 h TTX treatment, TNF-α induces potentiation of mEPSC amplitude associated with an increase in AMPA receptor surface expression level [25]. Based on the above observation, we suggest that the effect of IL-10 on HSP induction might be associated with the regulation of AMPA receptor activity. So we studied if co-incubation with IL-10 would affect neuronal [Ca2+]_i_ signals in response to the application of 5-Fluorowillardiine, a selective agonist of AMPA receptors. We found that 24 h co-incubation of IL-10 with TTX was able to effectively modulate a dose-dependent effect of 5-Fluorowillardiine on neuronal [Ca2+]_i,_, shifting the dose-dependent curve leftward and leading to a reduction in EC50 values and an increase in the Hill coefficient, thus resulting in neuronal sensitization to 5-Fluorowillardiine. Previously we have found that ~50 min incubation with IL-10 ameliorated hypoxia-induced downregulation of hippocampal AMPA GluR1 subunit and did not have any affect on GluR2 subunit expression level [9,10]. On the other hand, it was found that 24 h incubation with IL-10 exerts neuroprotective action via upregulation of AMPA receptor subunit GluR2 in cortical neurons of Sip1 mutant mice, an animal model of Mowat–Wilson syndrome [14]. Based on this, and our findings, we assume that 24 h co-incubation of IL-10 with TTX may exert similar effect inducing changes in AMPA receptors activity via modulation of their subunits expression level in cultured hippocampal neurons under normal conditions. Yet, further studies are needed to test this hypothesis.

It is critical to mention that the present study was performed with mixed neuroglial cultures. Previously it has been found that IL-10 receptors are expressed not only in neurons but also in astrocytes and microglia [26,27,28,29]. Both astrocytes and microglia are considered as important modulators of synaptic plasticity [30,31,32]. Thus, it will be critical in the future to elucidate if exogenous IL-10 affects synaptic transmission and HSP directly via the modulation of neuronal activity or if the effect is primarily attributed to its action on neuroglial IL-10 receptors.

**Figure 5:**
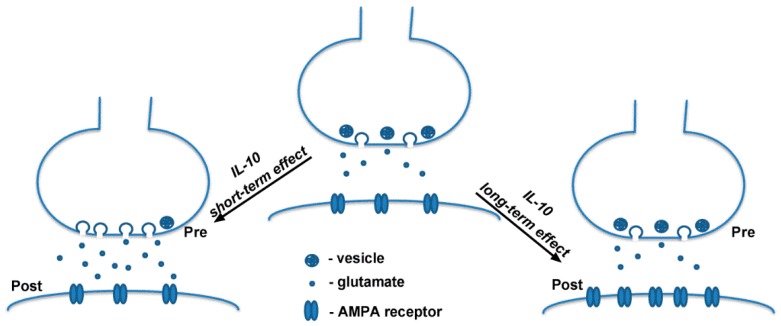
Working model of IL-10 action on synaptic activity. Short-term effect of IL-10 on synaptic activity might be associated with its action on probability of vesicle release at presynaptic site. Long-term effect of IL-10 might be related to its action on the AMPA receptor expression level at postsynaptic site.

In conclusion, we found that exogenous IL-10 differentially modulate glutamatergic synaptic activity at normal experimental conditions. We assume that the short-term effect of IL-10 might be associated with the potentiation of presynaptic vesicle release while long-term effect could be related to a postsynaptic increase in the AMPA receptors level (Figure 5). Obtained results show that IL-10, along with other interleukins, plays an important role in the regulation of synaptic activity and may be critical for normal cognitive function as impairment in IL-10 expression was found to be associated with the development of cognitive dysfunction in neurodegenerative disorders such as Alzheimer’s disease [33,34].

## 4. Material and Methods

### 4.1. Preparation of Primary Hippocampal Culture

Co-culture of hippocampal neurons and astrocytes isolated from the brains of newborn Sprague–Dawley rats (1 day old) were used in the present study. All animal studies were approved by the Animal Ethics Committees at the Institute of Theoretical and Experimental Biophysics RAS. Animals were quickly sacrificed by decapitation, then hippocampi were extracted and dissociated with clippers and incubated for 2 min in cold Hank’s balanced salt solution (HBSS) containing Ca^2+^ and Mg^2+^. Supernatant was removed with a pipette. Next, 2 mL of trypsin (0.1% in the Ca^2+^–Mg^2+^-free Hank’s solution) was added to the pellet to cover the whole tissue. The prepared mixture was incubated in a B27-supplemented neurobasal medium for 15 min at the 37 °C with constant mixing on a thermoshaker at 600 rpm. Then, trypsin was inactivated by equal volume of cold embryo serum, and the prepared mixture was centrifuged at 300× *g* for 5 min. To remove trypsin, cells were centrifuged in the neurobasal medium twice. Next, cells were re-suspended in the medium with an addition of glutamine (0.5 mM), B-27 supplement (2%) and gentamicin (20 g/mL). Then, 200 μL of suspension was added into a glass ring with an internal diameter of 6 mm residing on a poly-l-lysine covered round coverslip with a 25 mm diameter (VWR International, Radnor, PA, USA). Cells were transferred into the CO2-incubator and left at 37 °C for 5 h for attachment of the cells. After that, the cylinders were retrieved, and the culture medium was adjusted to 1.5 mL. The culture medium (2/3 of the total volume) was substituted with fresh portions on a daily basis. Neuroglial cell culture at an age between 13–15 days in vitro (DIV) were used in experiments with whole-cell patch clamp technique.

### 4.2. Whole-Cell Patch Clamp Recordings

Registration of miniature excitatory postsynaptic currents (mEPSC) from pyramidal neurons in primary neuroglial culture of hippocampus was carried out by means of whole-cell patch clamp. Pyramidal neurons were identified based on the soma morphology using a SliceScope (Scientifica, UK) equipped with a CCD camera. Recordings were done with a PC505B amplifier (Warner Instruments, Hamden, CT, USA) in voltage clamp mode. Signals were filtered at 2 kHz with the amplifier, then acquired and digitized at 10 kHz sampling frequency with ADC/DAC Digidata 1440A (Molecular Devices, USA), and the pClamp 10 software package for data acquisition and analysis (Molecular Devices, USA). Recording electrodes of 4–5 mOhm resistance were pulled from borosilicate glass capillaries (Harvard Apparatus, USA) using a PC10 vertical puller (Narishige, Japan). The composition of the intracellular solution used for electrode filling was as follows (in mM): 115 K-gluconate, 10 KCl, 2 Na_2_ATP, 10 Na_2_phosphocreatine, 1 MgCl_2_, 0.5 EGTA, 10 HEPES, pH 7.2, and osmolality of 280 ± 5 mOsm. Resistance of the electrodes was 4–5 MOhm. Hank’s solution used as an extracellular solution and contained (in mM): 139 NaCl, 4.17 NaHCO3, 0.4 NaH2PO4, 2.1 KCl, 0.44 KH2PO4, 2 CaCl_2_, 1.25 MgSO4, 5 HEPES, 8 d-glucose, pH 7.4, and osmolality 305 ± 2 mOsm. In addition, for mEPSC isolation from other synaptic events, 20 µM of bicuculine, in order to block GABAergic synaptic transmission, and 1µM of TTX to block action potential driven synaptic activity were added to the extracellular solution. All experiments were conducted at 27–28 °C. Access resistance was monitored throughout the recording and was typically <30 MΩ. Neurons which had an access resistance change of more than 15–20% during recording were excluded from the analysis.

### 4.3. Fluorescence Measurements of Neuronal Ca^2+^ Signals

The level of [Ca^2+^]_i_ in neurons was assessed by measuring fluorescence intensity of ratiometric Ca^2+^ sensitive probe using Fura-2 and analyzed, as previously described [14,35]. Briefly, neurons were loaded with Fura-2 dissolved in Hank’s balanced salt solution (HBSS) at a final concentration of 5 µM at 37 °C for 40 min with a subsequent 15 min washout. HBSS consisted of the following (mM): 156 NaCl, 3 KCl, 2 MgSO_4_, 1.25 KH2PO_4_, 1.4 CaCl_2_, 10 glucose and 10 HEPES, pH 7.4. Registration of fluorescent signals was made with a fluorescent system using an inverted fluorescence microscope Axio Observer Z1 equipped with a high-speed mono-chrome CCD camera Hamamatsu ORCA-Flash 2.8. Lambda DG-4 Plus illuminator (Sutter Instruments, Novato, CA, USA) was used as a source of excitation for fluorescence. To excite and record the Fura-2 fluorescence, we used a 21HE filter set (Carl Zeiss, Oberkochen, Germany) with the excitation filters BP340/30 and BP387/15, FT409 separator and BP510/90 emission filter.

### 4.4. Statistical Analysis

All values are given as mean ± SEM. All data were statistically analyzed with GraphPad Prism 7 (GraphPad Software, San Diego, CA, USA). Comparison of experimental groups was done with paired *t*-test or analysis of variance followed by Bonferroni correction for multiple comparisons or Kolmogorov–Smirnov test. P ≤ 0.05 was considered significantly different.
